# Pretransplant plasma soluble PD-1 and recurrence after liver transplantation in ICI-treated hepatocellular carcinoma: an exploratory biomarker study

**DOI:** 10.3389/fonc.2026.1944586

**Published:** 2026-10-15

**Authors:** Lizhen Zhu, Ruolin Wang, Wenzhi Shu, Yi Gao, Geng Zhang, Wenlong Zhao, Yucheng Hou, Qian Lu

**Affiliations:** 1 Qinghai University Affiliated Hospital (Clinical Medical College), Xining, China; 2 Hepatopancreatobiliary Center, Organ Transplantation Center, Beijing Tsinghua Changgung Hospital, School of Clinical Medicine,Tsinghua University, Beijing, China; 3 Beijing Key Laboratory of Liver Transplantation and Bionic Manufacturing, Beijing, China; 4 Institute for Organ Transplant and Bionic Medicine, Tsinghua University, Beijing, China; 5 Department of Hepatobiliary and Pancreatic Surgery, Affiliated Hospital of Qinghai University, Xining, China; 6 School of Clinical Medicine, Tsinghua University, Beijing, China; 7 Department of Orthopedics, Yingde People’s Hospital, Yingde/Qingyuan, Guangdong, China

**Keywords:** biomarker, hepatocellular carcinoma, immune checkpoint inhibitors, liver transplantation, recurrence, soluble PD-1

## Abstract

**Background:**

Immune checkpoint inhibitors (ICIs) are increasingly used as bridging or downstaging therapy before liver transplantation (LT) for hepatocellular carcinoma (HCC), but biomarkers of posttransplant recurrence in this setting are lacking. We evaluated the association between pretransplant plasma soluble programmed death-1 (sPD-1) and posttransplant outcomes after pretransplant ICI therapy.

**Methods:**

We enrolled 105 HCC patients undergoing LT, including 51 receiving ICIs and 54 without prior ICI exposure. Pretransplant plasma sPD-1 and sPD-L1 were measured by ELISA. The primary endpoint was 2-year recurrence-free survival (RFS). Associations were evaluated using Kaplan–Meier analysis and Cox regression.

**Results:**

Pretransplant sPD-1 was higher in the ICI group than in the non-ICI group (P < 0.0001). Within the ICI-treated cohort, patients who experienced recurrence had higher sPD-1 than those who did not (P = 0.0002), whereas sPD-L1 did not differ. At an internally derived exploratory cutoff of 2073 pg/mL, high sPD-1 was associated with lower 2-year RFS (7.7% vs. 76.3%; log-rank P < 0.0001; HR, 4.42; 95% CI, 1.75–11.17). As a continuous variable, each doubling of pretransplant sPD-1 was associated with an increased risk of recurrence in the unadjusted analysis (HR = 1.51, 95% CI 1.25–1.82; P < 0.001). The association remained significant after adjustment for AFP, tumor number, and maximum tumor diameter (adjusted HR = 1.51, 95% CI 1.20–1.89; P < 0.001).

**Conclusion:**

Elevated pretransplant sPD-1 is independently associated with increased posttransplant recurrence risk in HCC patients receiving ICIs before LT. This association remained after adjustment for AFP and pretransplant tumor burden, and appeared distinct from MVI status, ACR occurrence, and the timing of blood sampling. sPD-1 may serve as a complementary biomarker for risk stratification, but external validation is required.

## 1 Introduction

Liver transplantation (LT) remains the most effective curative treatment for patients with hepatocellular carcinoma (HCC) complicated by liver cirrhosis, and its indications are primarily based on tumor burden criteria such as the Milan criteria, University of California San Francisco (UCSF) criteria, and Hangzhou criteria (1, 2). For patients initially beyond these criteria, downstaging, conversion, or bridging therapies may enable requalification for LT (1, 3, 4). In recent years, immune checkpoint inhibitors (ICIs) targeting the programmed death-1 (PD-1)/programmed death-ligand 1 (PD-L1) pathway have demonstrated substantial efficacy in advanced HCC and have been increasingly applied as bridging or downstaging therapies prior to LT (5–10).

However, the use of ICIs in the pretransplant setting introduces new clinical challenges. Previous studies have primarily focused on safety concerns, particularly the risk of acute cellular rejection (ACR) after transplantation (11–13). Meanwhile, tumor recurrence may still occur after LT despite radiological response or successful downstaging following ICI-based therapy. Recent multicenter studies have demonstrated generally encouraging posttransplant survival but have also documented recurrent disease in a subset of patients, highlighting the need for improved recurrence-risk assessment in this emerging clinical setting (13–16). Established recurrence-risk indicators after LT include AFP, viable tumor burden, radiological or pathological response, and explant features such as MVI. These variables form the basis of validated posttransplant recurrence models but primarily characterize tumor burden and tumor biology rather than systemic immune status (17–19).

Soluble programmed death-1 (sPD-1) is a circulating isoform of membrane-bound PD-1 generated through proteolytic cleavage or alternative splicing, which may reflect peripheral T-cell activation and functional status (14, 20). Previous studies have suggested its potential prognostic value in various solid tumors (20–22). In patients receiving PD-1 blockade, circulating sPD-1 levels have also shown treatment-related changes and associations with treatment response or survival; however, the direction and magnitude of these associations have varied across tumor types, treatment regimens, sampling time points, and assay platforms (23–25). Its clinical significance in the specific context of HCC patients undergoing LT after ICI therapy therefore remains unclear.

Therefore, this study aimed to evaluate the association between pretransplant plasma sPD-1 levels and posttransplant recurrence risk in HCC patients receiving ICIs prior to LT. Given that circulating immune biomarkers may be influenced by treatment exposure and sampling timing, we additionally examined the relationships of sPD-1 with ICI treatment cycles and the interval between the last ICI administration and blood sampling.

## 2 Data and methods

### 2.1 Study design and participants

This retrospective cohort analysis was reported with reference to the STROBE statement and REMARK recommendations (26, 27). Patients with HCC who underwent LT at Beijing Tsinghua Changgung Hospital between January 2021 and June 2024 were assessed. The final cohort comprised 105 patients classified by pretransplant ICI exposure: 51 in the ICI group and 54 in the non-ICI group. The non-ICI reference group included 18 patients who proceeded directly to LT and 36 who received non-ICI bridging or downstaging therapy. These subgroups were combined because their baseline characteristics and pretransplant sPD-1 concentrations were comparable. **Figure 1** summarizes patient selection and the study timeline. All grafts were from deceased brain-dead donors. Center eligibility was primarily based on UCSF criteria; candidates beyond UCSF criteria were reviewed by a multidisciplinary team considering treatment response and other indicators of tumor biology.

**Figure 1:**
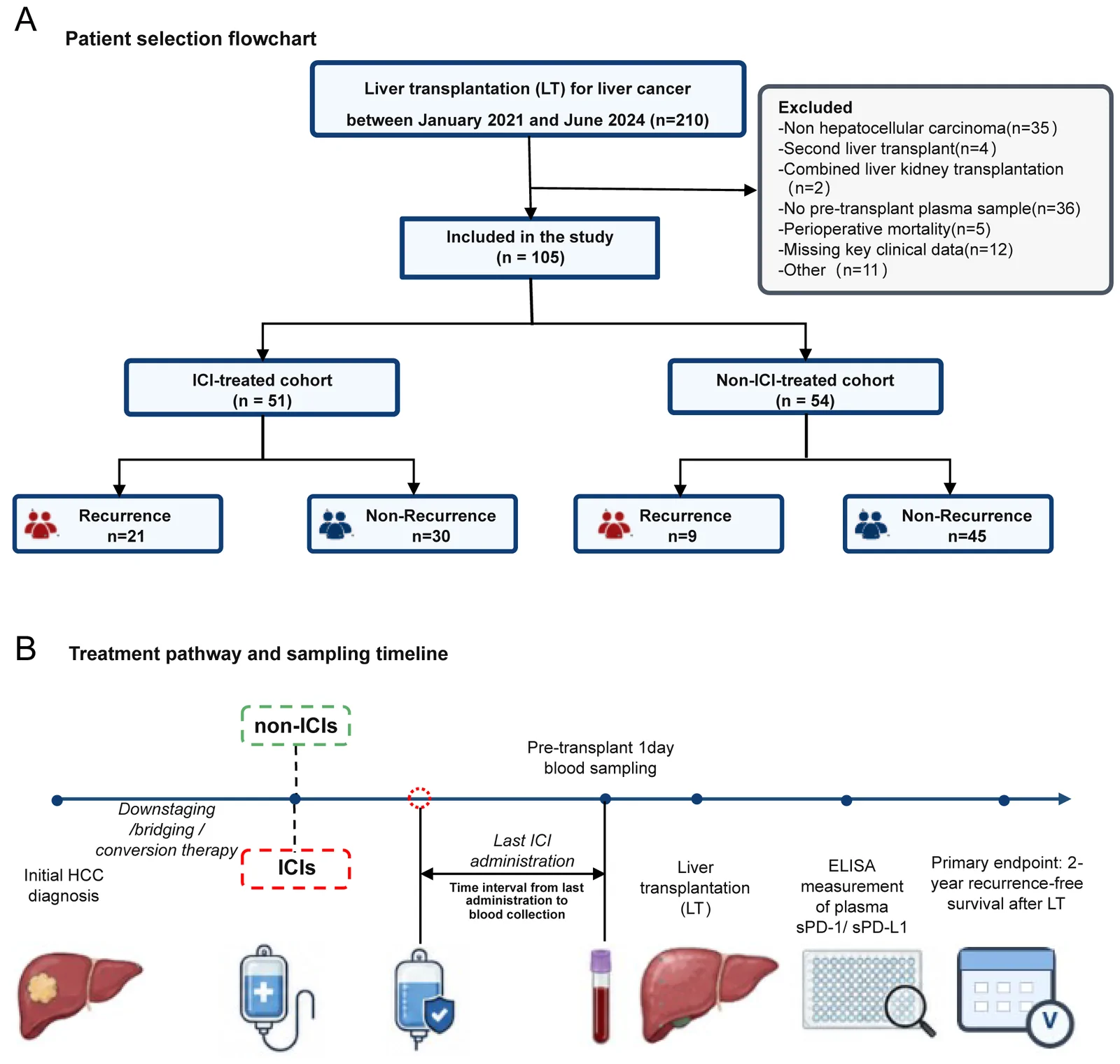
Study design and patient flow. **(A)** Flowchart of patient selection and grouping according to pretransplant ICI exposure. **(B)** Timeline of plasma sampling one day before LT and follow-up for recurrence-free survival (RFS).

The study was approved by the Ethics Committee of Beijing Tsinghua Changgung Hospital (Approval No. 22524-0-02) and registered as a clinical study (NCT05411926).

### 2.2 ICI treatment and response assessment

Patients in the ICI group received a PD-1 inhibitor, a PD-L1 inhibitor, or both; some also received targeted therapy, transarterial chemoembolization, hepatic arterial infusion chemotherapy, or radiotherapy. ICI agent, number of cycles, and the interval from the final ICI dose to blood sampling were recorded. Tumor response was assessed using modified Response Evaluation Criteria in Solid Tumors (mRECIST): complete or partial response was classified as response, and stable or progressive disease as non-response.

### 2.3 Transplant eligibility and surgery

LT eligibility and timing were determined by a multidisciplinary team considering established transplant criteria, tumor burden and treatment response, liver function, performance status, and donor availability. Patients initially beyond the center’s criteria could be reconsidered after bridging or downstaging therapy.

### 2.4 Plasma collection and ELISA

Peripheral blood was collected one day before LT. Plasma was separated by centrifugation, stored at −80 °C in a centralized biobank, and not subjected to repeated freeze–thaw cycles. sPD-1 and soluble PD-L1 were measured using the Human PD-1 Quantikine ELISA Kit (DPD10; R&D Systems, Minneapolis, MN, USA) and Human/Cynomolgus Monkey PD-L1/B7-H1 Quantikine ELISA Kit (DB7H10; R&D Systems), respectively, according to the manufacturer’s instructions. Samples were assayed in duplicate by laboratory personnel blinded to clinical outcomes. The stated analytical range of the sPD-1 assay was 15.6–1000 pg/mL; samples above the upper limit were diluted and reassayed. Raw concentrations are reported in pg/mL. Log10-transformed values were used for graphical comparisons and correlation analyses. In Cox models, sPD-1 was log2-transformed so that the hazard ratio represented the association with each doubling of concentration.

### 2.5 Clinical data collection

Clinical data were collected, including age, sex, underlying liver disease etiology, Model for End-Stage Liver Disease (MELD) score, tumor characteristics at initial diagnosis and immediately before transplantation, AFP, protein induced by vitamin K absence or antagonist-II (PIVKA-II), immunotherapy-related information, mRECIST response assessment, explant pathological findings, MVI status, tumor differentiation grade, ACR, and recurrence and survival outcomes. MVI was determined based on postoperative pathological examination and was not included as a covariate in pretransplant Cox regression models. Subgroup analyses stratified by MVI-negative status were performed as exploratory pathological stratification to evaluate the consistency of the sPD-1 signal in patients with conventionally low-risk pathological features.

### 2.6 Diagnosis and management of acute cellular rejection

ACR was diagnosed based on Banff criteria through histopathological evaluation of liver biopsy specimens. All patients with ACR received methylprednisolone pulse therapy and/or adjustments to immunosuppressive regimens, resulting in normalization of liver function tests, with no cases of fatal ACR observed. As ACR occurs after liver transplantation and represents a posttransplant event, it was not included as a baseline covariate in pretransplant Cox regression models to avoid time-dependent bias.

### 2.7 Follow-up and study endpoint

The primary endpoint was 2-year RFS, defined as the interval from LT to the first documented tumor recurrence or death from any cause. Patients alive without recurrence were censored at their last follow-up.

### 2.8 Statistical analysis

Continuous variables were expressed as mean ± standard deviation or median with interquartile range, as appropriate. Group comparisons were performed using the independent-samples t-test or Mann–Whitney U test. Categorical variables were presented as counts and percentages and compared using the chi-square test or Fisher’s exact test, as appropriate. Because soluble checkpoint concentrations were right-skewed, log10-transformed values were used for the graphical Welch tests; **Table 1** additionally reports P values from Welch tests of the untransformed concentrations to retain the prespecified descriptive analysis. Correlations were assessed using Spearman’s rank coefficient. Linear regression quantified the association between the last-ICI-to-sampling interval and log10(sPD-1), with the interval scaled per 30 days.

**Table 1:** Clinical, tumor, pathological, and posttransplant characteristics of the ICI-treated and non-ICI-treated cohorts.

Characteristics	Total (n=105)	ICI (n=51)	Non-ICI (n=54)	P value
Age, years	55.06 ± 7.37	52.67 ± 7.03	57.33 ± 6.95	*0.001*
Sex, male, n (%)	96 (91.4)	47 (92.2)	49 (90.7)	>0.99
MELD score	20.23 ± 10.21	21.82 ± 10.41	18.76 ± 9.80	0.116
Initial tumor burden				
Maximum tumor diameter, cm	3.7 (2.3-5.6)	5.1 (2.6-7.6)	3.0 (1.9-4.3)	*0.0002*
Tumor number	3 (1–4)	4 (1–4)	2 (1–4)	*0.016*
Pretransplant tumor burden				
Maximum tumor diameter	2.0 (0.9-3.4)	1.9 (0.6-3.75)	2.1 (1.1-3.1)	0.594
Tumor number	1 (1–3)	1 (1-3.5)	1 (1-2)	0.844
AFP, ng/mL	12.7 (2.8-104.5)	13.0 (2.8-220.9)	11.3 (2.8-59.8)	0.072
PIVKA-II, mAU/mL	109.7 (30.7-524.2)	249.7 (38.6-986.2)	55.2 (28.5-215.2)	0.181
sPD-1, pg/mL	372.0 (205.0-801.0)	753.0 (326.3-2073.5)	253.8 (165.9-419.4)	*0.002*
sPD-L1, pg/mL	144.2 (97.0-403.0)	201.1 (128.0-583.5)	109.2 (80.6-200.9)	*0.002*
Transplantation standards				0.528
Within UCSF	83 (79.0)	39 (76.5)	44 (81.5)	
Beyond UCSF	22 (21.0)	12 (23.5)	10 (18.5)	
Macrovascular invasion	4 (3.8)	1 (2.0)	3 (5.6)	0.618
Edmondson grading				*0.0001*
Poorly differentiated	13 (12.4)	11 (21.6)	2 (3.7)	
Moderately differentiated	60 (57.1)	26 (51.0)	34 (63.0)	
Well differentiated	14 (13.3)	1 (2.0)	13 (24.1)	
Unknown/not available	18 (17.1)	13 (25.5)	5 (9.3)	
Microvascular invasion				0.880
No	83 (79.0)	40 (78.4)	43 (79.6)	
Yes	22 (21.0)	11 (21.6)	11 (20.4)	
Involving the liver capsule	28 (26.7)	18 (35.3)	10 (18.5)	0.052
Satellite lesions	10 (9.5)	7 (13.7)	3 (5.6)	0.194
ACR, n (%)	10 (9.5)	8 (15.7)	2 (3.7)	0.048
Recurrence, n (%)	30 (28.6)	21 (41.2)	9 (16.7)	*0.006*

Continuous variables are presented as mean ± standard deviation or median (interquartile range), and categorical variables as n (%). For sPD-1 and sPD-L1, the table reports Welch-test P values for untransformed concentrations; **Figure 2A** presents the corresponding log10-scale comparison, so its P value differs.

An exploratory sPD-1 cutoff of 2073 pg/mL was selected within the ICI-treated cohort using the Youden index from a conventional ROC analysis of observed recurrence status. Because the cutoff was derived and evaluated in the same cohort, dichotomized analyses were considered descriptive and were not intended to establish a clinical threshold. The AFP cutoff of 400 ng/mL was prespecified according to the Hangzhou criteria.

RFS was estimated by the Kaplan–Meier method and compared using the log-rank test. The primary Cox exposure was continuous log2(sPD-1); the internally dichotomized analysis was secondary. To limit model complexity given 21 recurrences in the ICI-treated cohort, covariates were restricted *a priori* to clinically relevant pretransplant measures. Adjustment models included: (1) AFP; (2) tumor number; (3) maximum tumor diameter; (4) tumor number and maximum tumor diameter; and (5) AFP, tumor number, and maximum tumor diameter (primary adjusted model). No data-driven variable selection was used.

The proportional hazards assumption was evaluated using Schoenfeld residuals. Exploratory subgroup analyses were conducted among mRECIST responders and patients without microvascular invasion (MVI). Log-transformed sPD-1 levels were compared between patients with and without recurrence in these subgroups using Welch’s t-tests. The two corresponding nominal P values were treated as one testing family and adjusted using the Benjamini–Hochberg procedure. In patients with AFP ≤400 ng/mL, recurrence-free survival was compared between high- and low-sPD-1 groups defined by the internally derived cutoff of 2073 pg/mL using the Kaplan–Meier method and log-rank test. Subgroup Cox analyses, where performed, were considered exploratory. Conventional ROC curves for observed recurrence status compared AFP, sPD-1, and a logistic-regression combination of both markers.

Harrell’s concordance index was used to assess the discrimination of a parsimonious Cox model including continuous log2-transformed sPD-1 and AFP. This model was used specifically for exploratory internal performance assessment and was distinct from the additional Cox regression analyses presented in **Table 2**. Internal validation was performed using 1,000 bootstrap resamples to estimate optimism and obtain an optimism-corrected C-index. Given the limited number of recurrence events, formal calibration of a complex multivariable model was not performed because the resulting estimates were considered potentially unstable. ROC analyses were considered exploratory development-cohort analyses and were reported descriptively.

**Table 2:** Characteristics of ICI-treated patients according to posttransplant recurrence status.

Characteristics	Total (n=51)	Recurrence (n=21)	Non-recurrence (n=30)	P
Age, years	52.67 ± 7.03	51.52 ± 6.18	53.47 ± 7.46	0.326
Sex, male, n (%)	47 (92.2)	19 (90.5)	28 (93.3)	>0.99
MELD score	21.82 ± 10.41	24.50 ± 10.0	19.88 ± 10.28	0.139
Maximum tumor diameter, cm	1.9 (0.6-3.75)	3.0 (1.4-4.8)	1.3 (0-2.3)	*0.012*
Tumor number	1 (1.0-3.5)	1 (1.0-4.0)	1 (0-3.0)	0.102
Macrovascular invasion	1 (2.0)	1 (4.8)	0 (0)	>0.99
Transplantation standards				0.167
Within UCSF	39 (76.5)	14 (66.7)	25 (83.3)	
Beyond UCSF	12 (23.5)	7 (33.3)	5 (16.7)	
mRECIST				0.923
Response	44 (86.3)	18 (85.7)	26 (86.7)	
Non-response	7 (13.7)	3 (14.3)	4 (13.3)	
AFP, ng/mL	13.0 (2.8-220.9)	91.2 (15.0-549.1)	6.4 (2.1-68.3)	0.060
PIVKA-II, mAU/mL	249.7 (38.6-986.2)	226.9 (47.9-1910.9)	249.7 (27.4-560.5)	0.994
sPD-1, pg/mL	753.0 (326.3-2073.5)	4760.5 (667.0-12197.0)	470.9 (291.7-851.3)	*0.002*
sPD-L1, pg/mL	201.1 (128.0-583.5)	176.0 (108.0-527.0)	323.5 (137.5-585.8)	0.881
Edmondson grading				0.128
Poorly differentiated	11 (21.6)	4 (19.0)	7 (23.3)	
Moderately differentiated	26 (51.0)	13 (61.9)	13 (43.3)	
Well differentiated	1 (2.0)	0 (0)	1 (3.3)	
Unknown/not available	13 (25.5)	2 (9.5)	11 (36.7)	
Microvascular invasion				0.165
No	40 (78.4)	14 (66.7)	26 (86.7)	
Yes	11 (21.6)	7 (33.3)	4 (13.3)	
Pathological response				*0.003*
Yes	45 (88.2)	15 (71.4)	30 (100)	
No	6 (11.8)	6 (28.6)	0 (0)	
Type of ICI agent				>0.99
PD-1	41 (80.4)	17 (81.0)	24 (80.0)	
PD-L1	3 (5.9)	1 (4.8)	2 (6.7)	
PD-1+PD-L1	7 (13.7)	3 (14.3)	4 (13.3)	
Number of ICI treatment cycles	5 (4-10)	5 (4-6)	5 (3-10)	0.967
Last medication - testing time	65.5 (44.0-121.5)	75.0 (54.0-90.0)	62.5 (31.0-150.0)	0.542

Continuous variables are presented as mean ± standard deviation or median (interquartile range), and categorical variables as n (%).

Analyses were performed using GraphPad Prism 11.0, SPSS 20.0, and R 4.3.1. All tests were two-sided, and P < 0.05 was considered statistically significant. Except for the two specified subgroup-level comparisons adjusted using the Benjamini–Hochberg procedure, no formal multiplicity adjustment was applied to other secondary or exploratory analyses. These analyses should therefore be interpreted as exploratory and hypothesis-generating.

## 3 Results

### 3.1 Research design and patient population

A total of 105 patients with HCC undergoing LT were included in the final analysis, comprising 51 patients in the ICI group (21 with recurrence and 30 without recurrence) and 54 patients in the non-ICI group (9 with recurrence and 45 without recurrence) (**Figure 1A**). Baseline characteristics of the ICI and non-ICI groups are summarized in **Table 1**. At initial diagnosis, tumor burden was greater in the ICI-treated cohort than in the non-ICI-treated cohort. Immediately before transplantation, maximum tumor diameter and tumor number were comparable between the cohorts (all P > 0.05).

### 3.2 Posttransplant management and clinical course

Following LT, all patients received triple maintenance immunosuppression consisting of a calcineurin inhibitor (tacrolimus or cyclosporine), mycophenolate mofetil, and corticosteroids. During follow-up, biopsy-proven ACR occurred in 8 patients (15.7%) in the ICI group and 2 patients (3.7%) in the non-ICI group. According to the Banff grading system, 4 episodes were classified as mild and 6 as moderate. All episodes resolved after methylprednisolone pulse therapy and/or adjustment of immunosuppression; no ACR-related deaths occurred. Pretransplant sPD-1 did not differ significantly between patients with and without ACR (P > 0.05), although the small number of ACR events limited precision.

### 3.3 Characteristics according to recurrence status

Among the 51 patients in the ICI group, 21 developed tumor recurrence during follow-up. The clinical characteristics of patients with and without recurrence are summarized in **Table 3**.

**Table 3:** Cox regression analyses of recurrence-free survival in ICI-treated patients undergoing liver transplantation for HCC.

Characteristics	Univariable Cox regression			Multivariable Cox regression		
	HR	95% CI	P	HR	95% CI	P
Maximum tumor diameter, cm	1.179	1.057-1.316	0.003	–	–	–
Tumor number	1.08	0.81-1.45	0.587	–	–	–
Macrovascular invasion	2.079	0.842-5.136	0.113	–	–	–
MELD score	1.4	0.92-2.14	0.119			
MVI	3.087	1.201-7.937	0.019	–	–	–
AFP, ng/ml	1.001	1.000-1.002	0.002	1.057	1.012-1.103	0.033
sPD-1, pg/ml	1.006	1.002-1.010	0.005	1.005	1.000-1.010	0.043

**Table 2** presents additional exploratory Cox regression analyses and should not be interpreted as a separately validated prediction model.

### 3.4 ICI exposure and pretransplant soluble checkpoint levels

Pretransplant plasma sPD-1 was higher in the ICI group than in the non-ICI group (P < 0.0001; **Figure 2A**). Soluble PD-L1 also differed between groups (**Supplementary Figure S1**). These cross-sectional comparisons are compatible with altered circulating PD-1/PD-L1-related profiles after ICI exposure but cannot establish a causal treatment effect or biological source.

**Figure 2:**
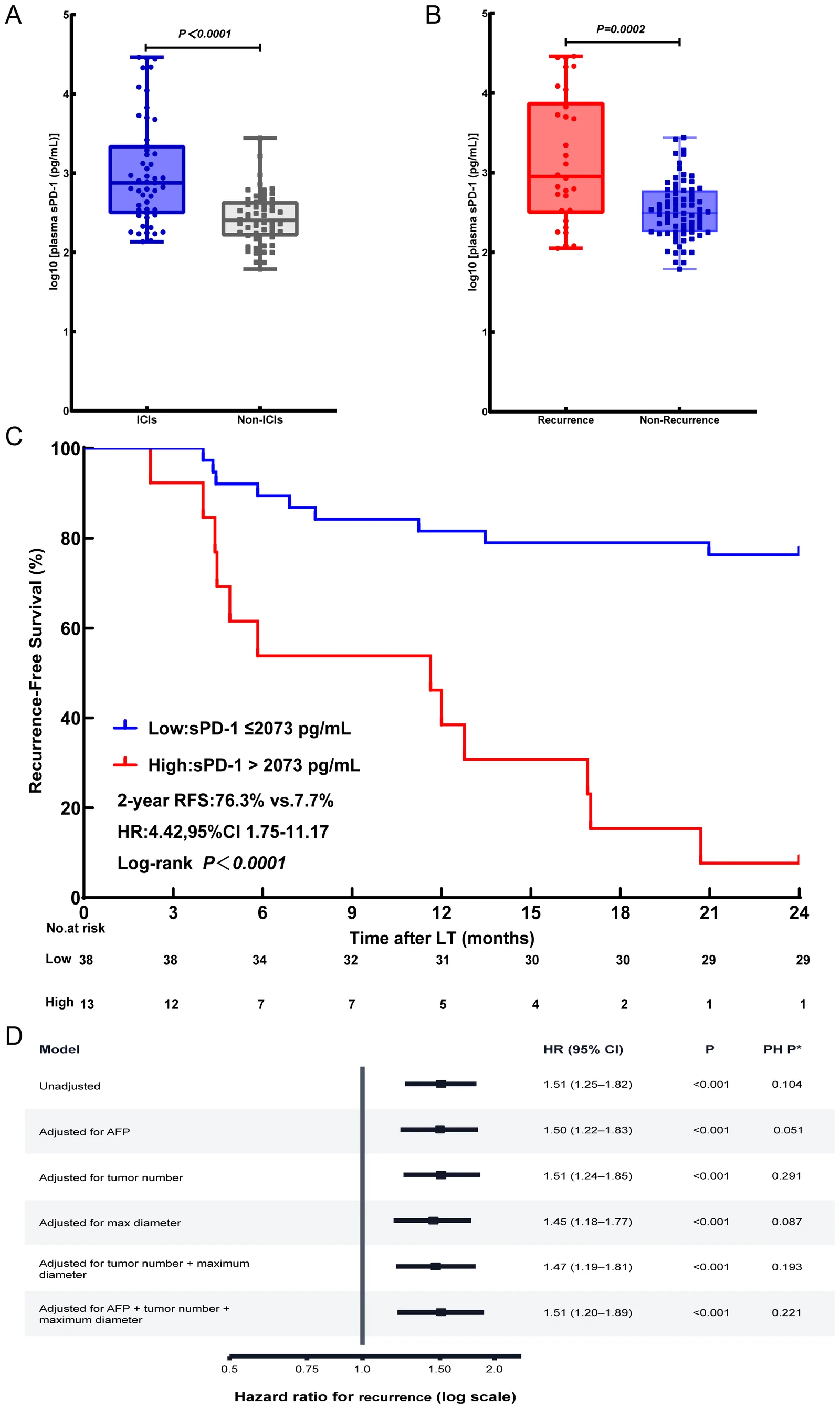
Association between pretransplant plasma sPD-1 and posttransplant recurrence. **(A)** Comparison of pretransplant plasma sPD-1 levels between the ICI and non-ICI groups. **(B)** Comparison of pretransplant plasma sPD-1 levels between recurrent and non-recurrent patients in the ICI-treated cohort. **(C)** Kaplan–Meier curves for 2-year RFS stratified using the exploratory sPD-1 cutoff of 2073 pg/mL. **(D)** Association between log2-transformed sPD-1 and recurrence across prespecified Cox regression models. PH P denotes the P value for the proportional hazards assumption based on Schoenfeld residuals.

### 3.5 Association of pretransplant sPD-1 with posttransplant recurrence in the ICI-treated cohort

Within the ICI-treated cohort, patients who experienced recurrence had higher pretransplant log10(sPD-1) than those who did not (P = 0.0002; **Figure 2B**). Soluble PD-L1 did not differ significantly according to recurrence status (**Supplementary Figure S2**). This comparison was exploratory given the limited number of events.

Using an exploratory cutoff of 2073 pg/mL (log10 ≈ 3.32) derived from the Youden index, patients were stratified into high and low sPD-1 groups. In the ICI-treated cohort, high sPD-1 levels were associated with significantly reduced 2-year recurrence-free survival (7.7% vs. 76.3%, log-rank P < 0.0001, **Figure 2C**) and increased risk of recurrence (HR = 4.42, 95% CI 1.75–11.17). When analyzed as a continuous variable, each doubling of pretransplant sPD-1 was associated with a 51% increase in the hazard of posttransplant recurrence in the unadjusted Cox model (HR = 1.51, 95% CI 1.25–1.82; P < 0.001). The effect estimates remained stable across the prespecified adjustment models, with hazard ratios ranging from 1.45 to 1.51. In the primary model adjusted for AFP, tumor number, and maximum tumor diameter, log2(sPD-1) remained associated with recurrence (adjusted HR = 1.51, 95% CI 1.20–1.89; P < 0.001; **Figure 2D**).

In separate univariable Cox analyses, maximum tumor diameter, MVI, AFP, and sPD-1 were associated with recurrence, whereas tumor number, macrovascular invasion, and MELD score were not. In the multivariable model including AFP and sPD-1, both biomarkers remained significantly associated with recurrence (**Table 2**). This model was distinct from the parsimonious model used for the exploratory C-index analysis.

For exploratory internal performance assessment, a parsimonious Cox model including continuous log2-transformed sPD-1 and AFP yielded an apparent Harrell’s C-index of 0.745. Based on 1,000 bootstrap resamples, the mean optimism was 0.009, yielding an optimism-corrected C-index of 0.736 (bootstrap 95% CI, 0.611–0.868). These estimates represent exploratory internal performance in the ICI-treated cohort and do not substitute for external validation.

### 3.6 Exploratory analyses in conventionally lower-risk subgroups

We explored whether pretransplant sPD-1 stratified residual RFS risk among patients with favorable conventional features (**Figure 3**).

**Figure 3:**
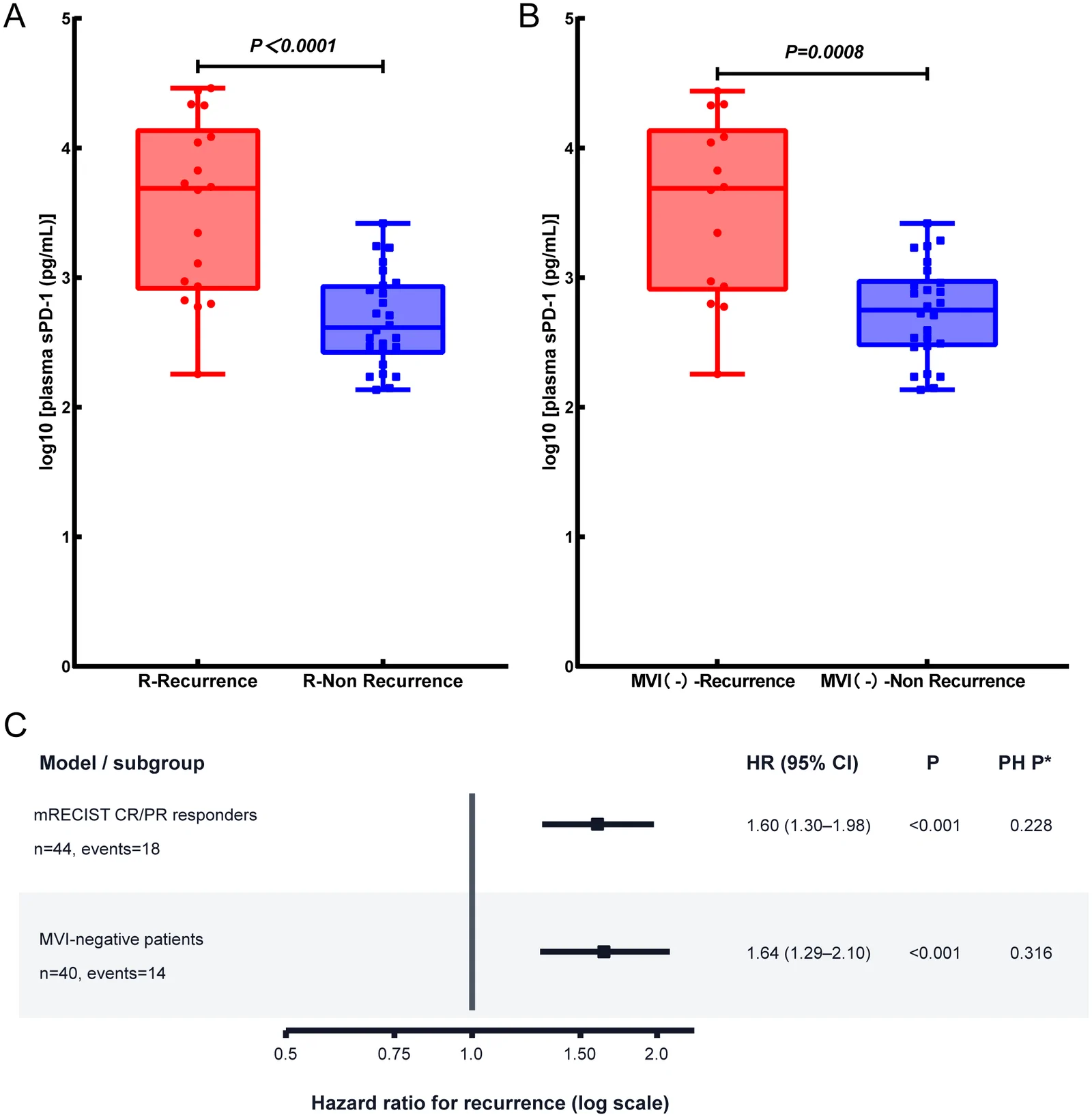
Exploratory subgroup analyses of sPD-1. **(A)** sPD-1 according to recurrence status among mRECIST responders. **(B)** sPD-1 according to recurrence status among patients without MVI. **(C)** Cox estimates per doubling of sPD-1 in the subgroups. PH P denotes the P value for the proportional hazards assumption based on Schoenfeld residuals.

Among mRECIST responders, patients who experienced recurrence had higher pretransplant log10(sPD-1) than those who did not (P = 0.00009; BH-adjusted P = 0.00018; **Figure 3A**). Each doubling of sPD-1 was associated with a higher hazard of an RFS event in the subgroup Cox analysis (HR, 1.60; 95% CI, 1.30–1.98; P < 0.001; **Figure 3C**).

Similarly, in the MVI-negative subgroup, recurrent patients also showed significantly elevated pretransplant sPD-1 levels compared with non-recurrent patients (P = 0.008; BH-adjusted P = 0.008; **Figure 3B**). The association between log2(sPD-1) and recurrence risk remained significant in Cox analysis (HR = 1.64, 95% CI 1.29–2.10; P < 0.001, **Figure 3C**). Given that MVI is a posttransplant pathological feature, this subgroup analysis was exploratory and intended to evaluate the robustness of the sPD-1 signal rather than to establish a preoperative predictive model.

These concordant exploratory findings suggest that sPD-1 may capture information not fully represented by radiological response or MVI status. They should not be interpreted as confirmatory subgroup effects because of the small sample size, limited number of recurrence events, internally derived cutoff, and absence of external validation.

### 3.7 ICI exposure characteristics, sampling interval, and sPD-1

To evaluate whether the timing of blood sampling influenced circulating sPD-1 levels, we analyzed the relationship between sPD-1 and both the number of ICI treatment cycles and the interval between the last ICI administration and blood collection (**Figure 4**).

**Figure 4:**
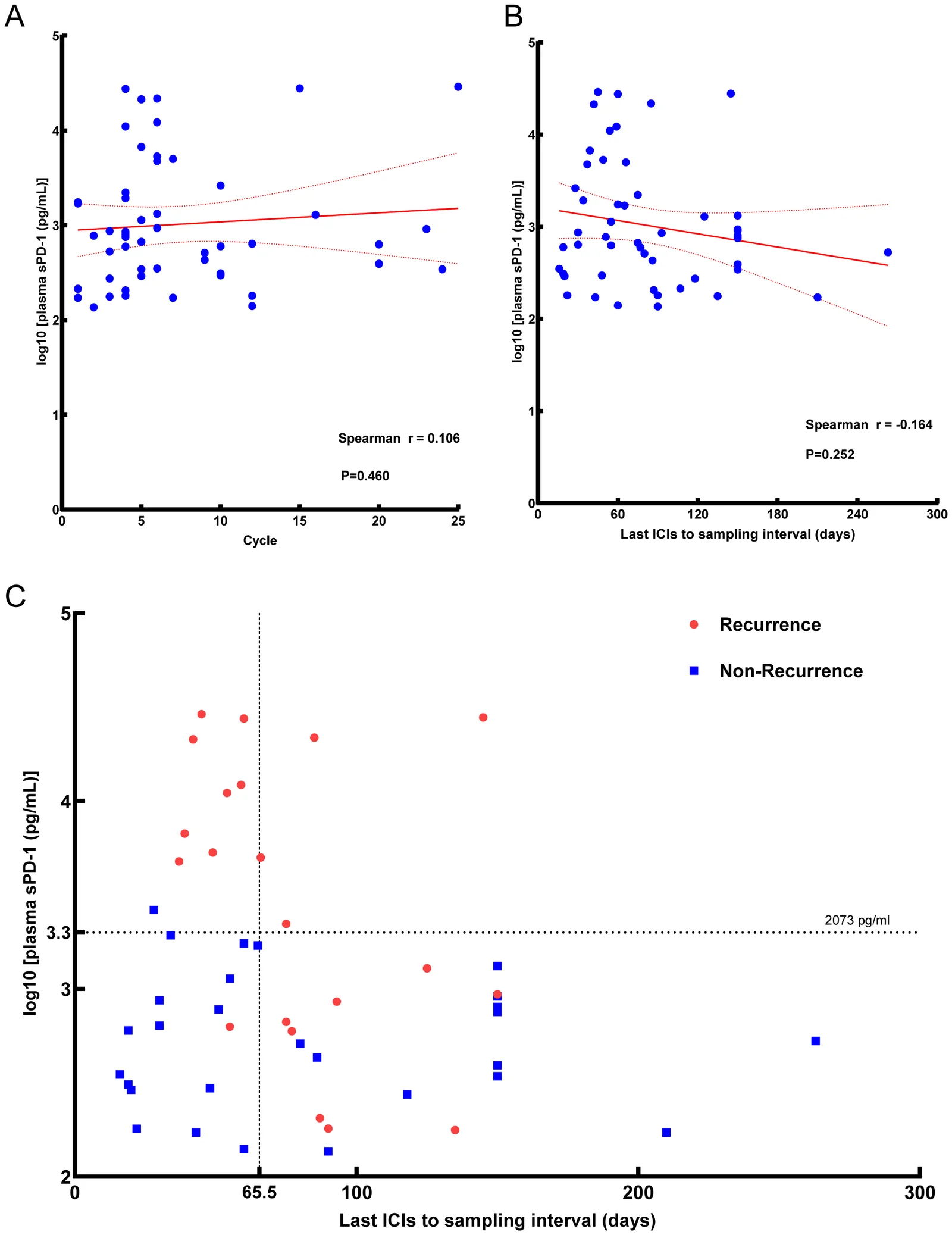
ICI exposure characteristics, sampling interval, and sPD-1. **(A)** Correlation between the number of ICI cycles and sPD-1. **(B)** Association between the last-ICI-to-sampling interval and sPD-1. **(C)** Joint distribution of sampling interval, sPD-1, and recurrence status. Dotted lines indicate the exploratory sPD-1 cutoff (2073 pg/mL) and median sampling interval (65.5 days).

No significant correlation was observed between the number of ICI treatment cycles and sPD-1 levels (Spearman r = 0.106, P = 0.460; **Figure 4A**). The interval between the last ICI administration and blood sampling showed a weak negative trend with sPD-1 levels, although this did not reach statistical significance (Spearman r = −0.164, P = 0.252; **Figure 4B**).

The median interval from the final ICI dose to blood sampling was 65.5 days. A joint display of interval, sPD-1, and recurrence status showed observations across both shorter and longer intervals (**Figure 4C**). This descriptive pattern did not reveal obvious clustering sufficient to explain the sPD-1 association, although it cannot rule out confounding by timing.

These analyses did not identify a statistically significant relationship between sampling interval and sPD-1, but the imprecision of the estimates precludes exclusion of a timing effect. Future studies should use standardized sampling windows and longitudinal measurements.

### 3.8 Exploratory complementary information from sPD-1 and AFP

Within the ICI-treated cohort, sPD-1 was not significantly correlated with AFP (Spearman r = 0.173; P = 0.226; **Figure 5A**), suggesting—but not proving—that the markers may contain partly distinct information. Patients with AFP > 400 ng/mL had lower 2-year RFS than those with AFP ≤ 400 ng/mL (33.3% vs. 66.7%; log-rank P = 0.018; **Figure 5B**), and elevated AFP was associated with a higher RFS event rate (HR, 3.39; 95% CI, 1.31–8.80).

**Figure 5:**
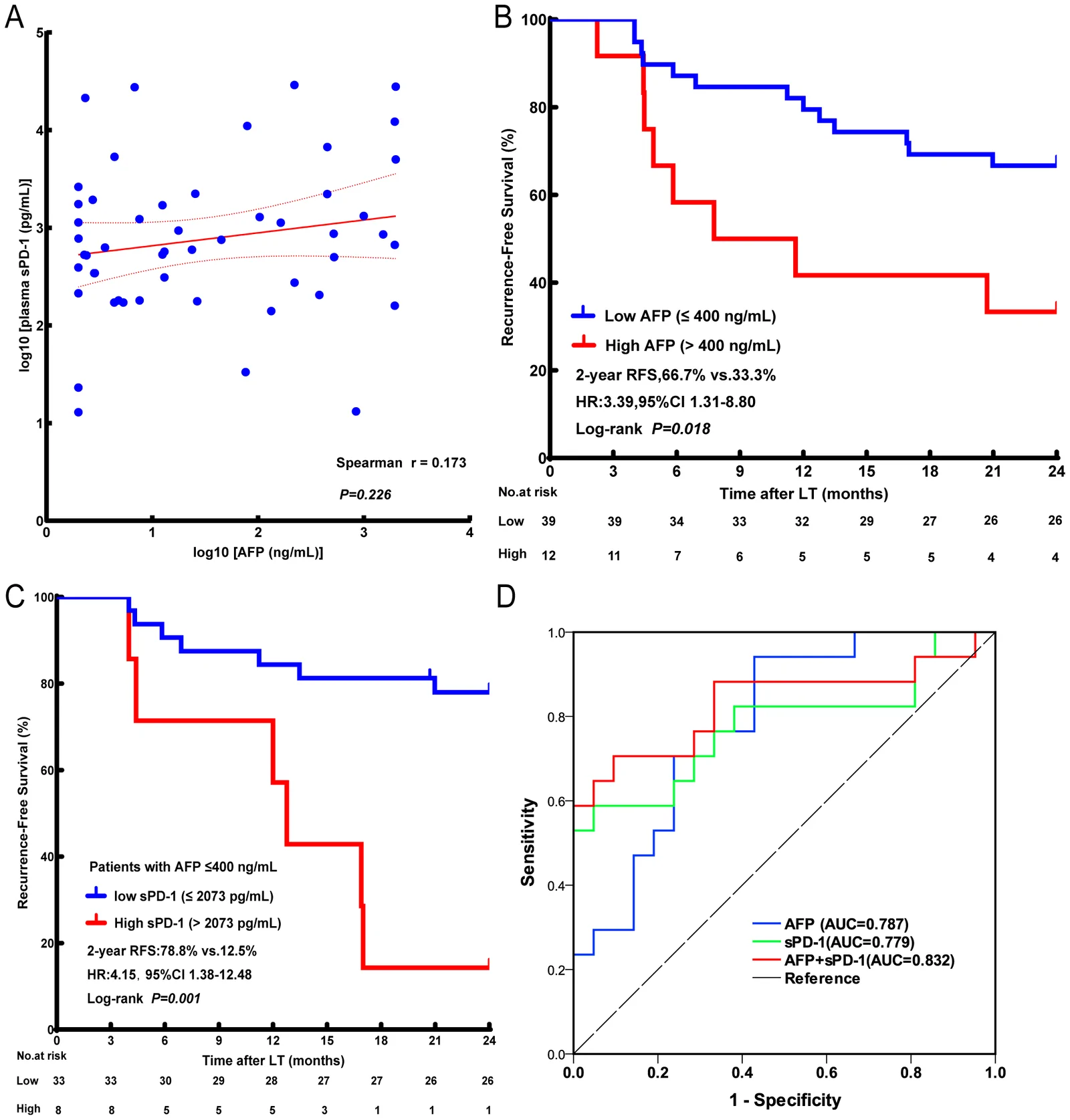
Complementary prognostic information provided by sPD-1 and AFP. **(A)** Correlation between log10-transformed sPD-1 and AFP levels. **(B)** Kaplan–Meier curves stratified by the predefined AFP cutoff of 400 ng/mL. **(C)** Kaplan–Meier curves stratified by the exploratory sPD-1 cutoff of 2073 pg/mL among patients with AFP ≤400 ng/mL. **(D)** ROC curves showing the discriminatory performance of AFP, sPD-1, and their combined model.

In patients with AFP ≤ 400 ng/mL, further stratification by the exploratory sPD-1 cutoff (2073 pg/mL) revealed that high sPD-1 levels were still associated with significantly poorer recurrence-free survival (log-rank P = 0.001; **Figure 5C**). Consistently, high sPD-1 remained significantly associated with increased recurrence risk (HR = 4.15, 95% CI 1.38–12.48). Given the limited sample size in this subgroup, particularly the small number of high sPD-1 cases, these findings should be interpreted as exploratory and require validation in larger cohorts.

In the exploratory conventional ROC analysis of observed recurrence status, the AUC was 0.787 for AFP, 0.797 for sPD-1, and 0.832 for the combined model (**Figure 5D**). Because this analysis ignored follow-up time and censoring and was conducted in the development cohort, the numerical AUC increase cannot establish incremental prognostic value.

## 4 Discussion

This study explored the association of pretransplant plasma sPD-1 in HCC patients undergoing LT after ICI therapy. The principal finding is that elevated pretransplant sPD-1 levels are independently associated with an increased risk of posttransplant tumor recurrence, and this association remains robust after adjustment for AFP and tumor burden. These results extend the focus of ICI-bridged liver transplantation from perioperative safety alone to risk stratification for tumor recurrence, providing exploratory evidence for a circulating immune-related biomarker in this emerging clinical setting.

Prior literature on ICI use before LT has emphasized allograft safety, particularly ACR (15). In the present cohort, the incidence of ACR in the ICI-treated group was 15.7%, consistent with previously reported trends, and all episodes were successfully managed without mortality (11–13, 28). We found no evidence of an association between pretransplant sPD-1 and ACR, but the small number of ACR events limits inference. Beyond allograft safety, recurrence after pretransplant ICI therapy remains incompletely characterized: multicenter cohorts and pooled analyses report generally favorable survival but persistent recurrence risk and relatively limited follow-up (11, 13, 15, 16). In our exploratory analyses, the sPD-1 association was concordant among mRECIST responders, patients without MVI, and patients with AFP ≤ 400 ng/mL. These findings raise the possibility that conventional tumor measures do not fully capture immune-related risk, but the subgroup estimates require independent confirmation.

The interpretation of circulating sPD-1 during or after ICI therapy remains uncertain. ICI-treated cohorts have reported heterogeneous associations of baseline or on-treatment sPD-1 with response and survival, consistent with effects of tumor type, immune context, regimen, sampling time, and assay platform (23–25). Established post-LT recurrence models incorporate AFP, viable tumor burden, and pathological features such as MVI, while additional circulating biomarkers, including AFP-L3 and DCP, can further refine recurrence-risk assessment (19, 29). Plasma-based molecular markers have also shown potential for postoperative recurrence surveillance, supporting the broader concept that circulating biomarkers may complement conventional clinicopathological variables (29). In the present study, the absence of a significant correlation between sPD-1 and AFP suggests that these biomarkers may capture partly distinct biological information. Accordingly, sPD-1 may provide complementary prognostic information beyond conventional tumor-related indicators. Although the combined model showed numerically improved discriminative performance in ROC analysis, this observation is exploratory and requires external validation.

Circulating immune biomarkers should be interpreted in the context of prior treatment exposure and the timing of biospecimen collection. In the setting of pretransplant ICI therapy, the interval between the last ICI administration and blood sampling may affect circulating immune-marker levels and their clinical interpretation. In the present cohort, neither the number of ICI treatment cycles nor the interval between the last ICI administration and blood collection was significantly associated with pretransplant sPD-1 levels. These findings provide limited indirect reassurance that the observed association was not solely explained by differences in treatment exposure or sampling timing; however, potential residual confounding and assay interference cannot be excluded.

This study has several limitations. First, its single-center retrospective design, limited sample size, and 21 recurrence events restrict the precision and generalizability of the findings. Formal calibration of a complex multivariable model was not performed because of the limited number of events. The C-index and bootstrap estimates reflect exploratory internal performance and cannot replace external validation.

Second, the sPD-1 cutoff was internally derived and has not been externally validated. MVI was assessed posttransplant and was therefore unsuitable for pretransplant predictive modeling; the MVI-negative subgroup analysis was exploratory. In addition, the absence of immune-cell profiling, cytokine measurements, tumor-tissue immunophenotyping, and functional experiments limits mechanistic interpretation. The findings should therefore be interpreted as clinical associations rather than evidence that sPD-1 directly mediates posttransplant recurrence.

Finally, potential assay interference from residual therapeutic antibodies cannot be excluded. The lack of significant associations between sPD-1 and either the number of ICI treatment cycles or the last-ICI-to-sampling interval provides only limited indirect reassurance. Because the binding epitopes of the DPD10 antibodies and drug-specific interference data are unavailable, future studies should incorporate drug-specific interference testing, including spike-and-recovery, dilution-linearity, and antibody-blocking or competition experiments, together with orthogonal assay methods.

In conclusion, higher pretransplant plasma sPD-1 was associated with an increased risk of posttransplant recurrence in patients with HCC who received ICIs before LT. The effect estimate remained stable after adjustment for AFP and pretransplant tumor burden and was directionally consistent in exploratory clinically relevant subgroups. These findings support further evaluation of sPD-1 as an immune-related prognostic signal but remain hypothesis-generating. Independent cohort validation and direct assessment of potential therapeutic-antibody interference are required before clinical application.

## Data Availability

The original contributions presented in the study are included in the article/**Supplementary Material**. Further inquiries can be directed to the corresponding author.
